# Animal and plant protein intake association with mental health, tryptophan metabolites pathways, and gut microbiota in healthy women: a cross-sectional study

**DOI:** 10.1186/s12866-024-03534-8

**Published:** 2024-10-07

**Authors:** Neda Soveid, Bahareh Barkhidarian, Mahsa Samadi, Mahsa Hatami, Fatemeh Gholami, Mir Saeid Yekaninejad, Ahmad Saedisomeolia, Maryam Karbasian, Seyed Davar Siadat, Khadijeh Mirzaei

**Affiliations:** 1 Department of Community Nutrition, School of Nutritional Sciences and Dietetics, Tehran University of Medical Sciences (TUMS), P.O Box 6446, Tehran, 14155 Iran; 2 Department of Clinical Nutrition, School of Nutritional Sciences and Dietetics, Tehran University of Medical Sciences, Tehran, Iran; 3 Food Health Research Center, Hormozgan University of Medical Sciences, Bandar Abbas, Iran; 4 Department of Epidemiology and Biostatistics, School of Public Health, Tehran University of Medical Science, Tehran, Iran; 5 College of Health Sciences, Education Centre of Australia, Parramatta, NSW 2153 Australia; 6 School of Human Nutrition, McGill University, Montreal, Canada; 7 Department of Mycobacteriology and Pulmonary Research, Pasteur Institute of Iran, P.O Box 6446, Tehran, 14155 Iran; 8 Food Microbiology Research Center, Tehran University of Medical Sciences, Tehran, Iran

**Keywords:** Mental health, Depression, Anxiety, Stress, Gut microbiota, Protein source

## Abstract

Mental health is affected by tryptophane (TRP) metabolism regulation. Diet-influenced gut microbiome regulates TRP metabolism. Thus, the present study aimed to explore the relationship between type of dietary protein intake, gut microbiota, TRP metabolites homeostasis, and mental well-being in healthy women. 91 healthy females aged 18–50 were recruited based on the study protocol. Validate and reliable questionnaires assessed dietary intake and mental health. Biochemical tests and gut microbiota composition were analyzed following the manufacturer’s instructions for each enzyme-linked immune sorbent assay (ELISA) kit and Real-time quantitative polymerase chain reaction (qPCR) methods respectively. Regression methods were used to estimate the considered associations. The results show that in the fully adjusted model, plant protein consumption was partially inversely associated with depression risk (OR = 0.27; 95% CI: 0.06, 1.09; *P* = 0.06). Higher dietary animal protein intake was marginally associated with psychological distress (OR = 2.59; 95% CI: 0.91, 7.34; *P* = 0.07). KYN to serotonin ratio was inversely associated with animal protein consumption (ß = 1.10; 95% CI: -0.13, 2.33; *P* = 0.07). *Firmicutes/Bacteriodetes* ratio (β = -1.27 × 103, SE = 5.99 × 102, *P* = 0.03) was lower in the top tertile of plant protein. A partially negative correlation was found between dietary animal protein and *Prevotella* abundance (β = -9.20 × 1018, SE = 5.04 × 1018, *P* = 0.06). Overall, significant inverse associations were found between a diet high in plant protein with mental disorders, KYN levels, and *Firmicutes* to *Bacteroidetes* ratio while adhering to higher animal protein could predispose women to psychological stress.

## Introduction

A mental disorder is a syndrome of significant disturbance in an individual’s cognition, emotion regulation, or behavior, resulting in dysfunction of psychological, biological, or developmental processes [1]. They have a significant impact on millions of people. Depression, anxiety, and stress are among the most common complications. It is estimated that 4.7% of the world’s population suffers from depression, and 7.3% suffer from anxiety [2]. Mental health disorders are becoming more prevalent, and women are twice as likely to experience these conditions [3]. The mental health conditions in Iran also exhibit a similar pattern [4]. The impact of psychological disorders can vary from inadequate work and academic and social environments to susceptibility to chronic health conditions such as cancer, diabetes, and cardiovascular disease [5]. Therefore, finding the best approach to manage or control these disorders is important. Although the exact mechanism of mental disorders remains uncertain, genetic and environmental factors seem to play pivotal roles [6]. The identification of risk factors that modify the disease can be a useful tool for both prevention and management. Studies suggest that dietary intake components are involved in the formation, progression, and duration of mental health disorders [7]. One important part of a healthy balanced diet is protein. It can be obtained from both plant and animal foods. Plant sources were previously considered incomplete protein sources. However, recent findings indicate that a well-designed vegetarian diet can contain all the essential amino acids [8]. Amino acids (mainly TRP, phenylalanine, and tyrosine) are major nutrients that are believed to play important roles in mental health, contributing to the production of neurotransmitters [9]. Regulation of metabolism of the essential amino acid TRP plays an important role in brain-endocrine-immune system interactions thought to be involved in mental health [10]. Two main pathways metabolize TRP: the KYN pathway, which is initiated by the enzyme indoleamine-2,3-dioxygenase (IDO), and the serotonin pathway, which is undertaken by TRP hydroxylase [11]. The KYN pathway is the main TRP metabolic pathway, and most TRP (i.e., 99%) in the diet is metabolized by the KYN pathway [12]. Immunological challenges such as pro-inflammatory cytokines (IL-1, IL-6, and TNF-α) induce IDO activity, promote the KYN pathway, deprive the 5-HT pathway of TRP, and reduce 5-HT synthesis [13]. Moreover, the presence of a common transporter that crosses the blood–brain barrier results in competition between KYN and TRP [14]. Therefore, dysregulation of the balance between metabolites of the KYN and serotonin pathways is associated with an increased risk of stress, anxiety, and depression [15].

**Fig. 1:**
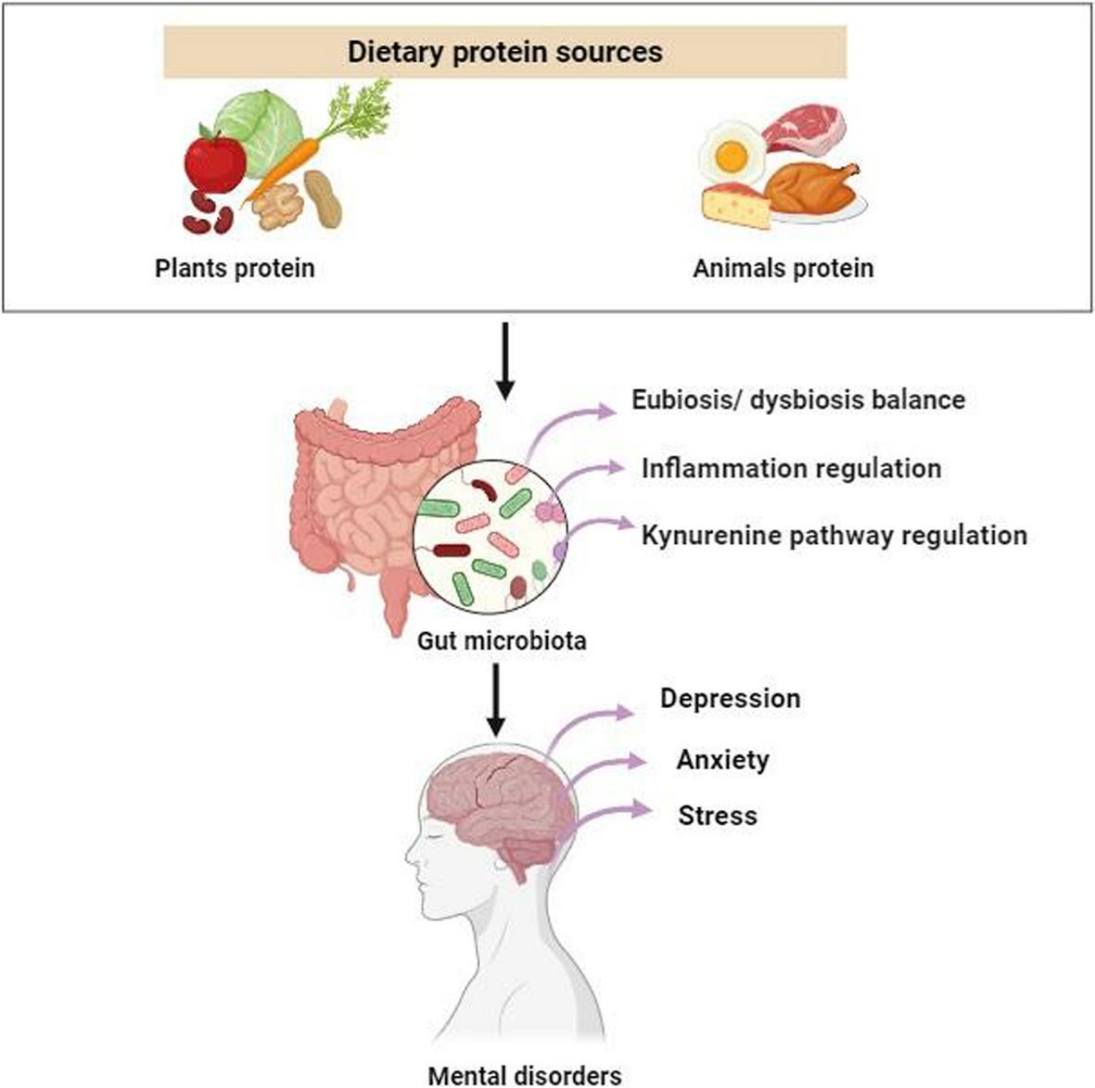
The association between dietary protein source and gut-brain axis. The figure illustrates how diet, gut microbiota composition, and mental disorders are interconnected. It suggests that dietary choices may significantly impact the management of mood disorders by affecting the balance of gut microbiota composition, which in turn affects TRP metabolism through the regulation of inflammation factors and the kynurenine pathway

The gut microbiota composition, which is affected by diet, can influence IDO activity and the regulation of TRP metabolism in several ways [16]. Therefore, the state of the gut microbiota could be an important determinant of serotonin homeostasis and mental health [17]. In recent studies, protein intake was positively correlated with overall gut microbial balance and diversity [18]. Furthermore, the metabolic activities of the gut microbiota could be affected by the type of dietary proteins of both plants and animals (Fig. 1) [19]. Animal proteins are considered a complete source of proteins, providing all amino acids. In contrast, people who consume only animal proteins are more likely to develop chronic inflammatory conditions and gut microbiota dysbiosis, but this is not the case for nutrient-providing plant proteins such as beta-carotene, fiber, vitamin C, vitamin E, folic acid, iron, magnesium, and calcium [20]. Although each protein source has advantages and disadvantages, more research is needed to determine its overall health impact. For the first time, the present study aimed to determine the associations between the type of dietary protein intake, the abundance of some bacteria and phyla, KYN and serotonin, which are TRP metabolites; IL-6, an inflammatory marker; and mental well-being in healthy women without mood disorders by conducting a cross-sectional study.

## Results

### Study population characteristics

Ninety-one participants completed the study protocol. The mean (SD) age, weight, and BMI of the participants were 35.87 (6.57) years, 70.53 (11.69) kg, and 27.03 (4.28) kg/m^2^, respectively. In terms of economic status, marital status, and employment status, 55 (60.4%) respondents had a moderate economic status, 47 (51.6%) respondents were married, and 48 (52.7%) participants were employed. The most of respondents were educated with a bachelor’s degree or higher (67 (73.6%)), while 39.6% of the participants were stressed, 39.6% were depressed and 34.1% were anxious.

### General characteristics of participants across the tertiles of dietary plant and animal protein

The baseline characteristics of the participants according to tertiles of dietary plants and animal protein are presented in Table 1. Individuals with a higher score for animal protein were older (*P* = 0.007). Compared with those in the bottom tertiles, individuals in the top tertiles of animal protein had a lower WHR (*P* = 0.04).

**Table 1:** General characteristics of study participants across the tertiles of dietary plant and animal protein

Variables	Tertiles of plant protein					Tertiles of animal protein				
	**T1** ** ≤ 24.81** **(*N* = 30)**	**T2** **24.82- 36.07** **(*N* = 31)**	**T3** ** ≥ 36.08** **(*N* = 30)**	*P-* value^*^	***P-* value**^**b**^	**T1** ** ≤ 21.69** **(*N* = 30)**	**T2** **21.70- 36.32** **(*N* = 31)**	**T3** ** ≥ 36.33** **(*N* = 30)**	*P-* value^*^	***P*- value**^**b**^
Age (year)	34.83 ± 5.74	35.87 ± 8.04	36.90 ± 5.64	0.48	0.28	33.30 ± 5.58	35.74 ± 5.71	38.57 ± 7.39	**0.007**	**0.008**
Weight (Kg)	70.42 ± 11.95	72.95 ± 12.22	68.15 ± 10.71	0.27	0.97	69.68 ± 11.57	72.55 ± 11.03	69.30 ± 12.55	0.49	0.33
BMI (Kg/m^2)^)	26.86 ± 4.09	27.93 ± 4.57	26.27 ± 4.12	0.30	0.47	26.64 ± 4.47	27.42 ± 4.26	27.03 ± 4.22	0.78	0.79
Physical activity (MET min-week)	1119.33 ± 1567.51	773.16 ± 865.59	1406.03 ± 2560.44	0.39	0.62	960.07 ± 885.05	1328.90 ± 2561.28	991.03 ± 1530.11	0.67	0.55
**Body composition**										
WC (cm)	90.80 ± 10.95	93.11 ± 11.71	89.21 ± 9.63	0.37	0.89	90.10 ± 10.47	93.04 ± 11.14	89.99 ± 10.88	0.46	0.08
WHR	0.90 ± 0.05	0.91 ± 0.05	0.90 ± 0.04	0.85	0.42	0.90 ± 0.04	0.92 ± 0.06	0.89 ± 0.04	0.19	**0.04**
BFM (Kg)	26.84 ± 8.56	29.90 ± 9.32	26.89 ± 8.02	0.29	**0.03**	27.70 ± 9.04	28.70 ± 8.13	27.27 ± 9.09	0.80	0.67
BF (%)	37.27 ± 6.44	39.74 ± 7.01	38.47 ± 6.34	0.34	**0.07**	38.53 ± 6.97	38.79 ± 5.75	38.20 ± 7.25	0.94	0.88
SMM (Kg)	23.79 ± 2.75	23.89 ± 3.39	22.34 ± 3.05	0.09	0.23	22.81 ± 2.56	24.02 ± 2.94	23.18 ± 3.74	0.30	0.51
FFM (%)	43.59 ± 4.62	43.85 ± 5.65	40.46 ± 6.76	**0.04**	0.11	42.44 ± 4.08	44.05 ± 4.86	41.39 ± 7.91	0.20	0.30
FMI	10.29 ± 3.33	11.79 ± 3.96	10.86 ± 3.34	0.25	**0.05**	11.15 ± 3.97	10.88 ± 3.23	10.95 ± 3.61	0.95	0.19
FFMI	16.66 ± 1.37	16.78 ± 1.70	15.93 ± 1.57	**0.07**	**0.03**	16.27 ± 1.49	16.61 ± 1.66	16.50 ± 1.62	0.69	0.98
**Blood pressure**										
SBP (mmHg)	103.56 ± 23.44	98.03 ± 26.83	99.46 ± 13.47	0.60	0.46	100.73 ± 22.11	103.25 ± 14.28	96.86 ± 27.81	0.52	0.10
DBP (mmHg)	80.83 ± 13.69	72.63 ± 15.61	74.23 ± 9.54	**0.04**	**0.04**	76.70 ± 10.68	77.81 ± 11.48	73.03 ± 17.57	0.36	0.19
**Marital status (n (%))**										
Single	11 (29.7)	17 (45.9)	9 (24.3)	0.09	**0.01**	13 (27.7)	20 (42.6)	14 (29.8)	0.10	0.53
Married	18 (38.3)	10 (21.3)	19 ( 40.4)			16 (43.2)	7 (18.9)	14 (37.8)		
Divorced/widow	1 (14.3)	4 (57.1)	2 (28.6)			1 (14.3)	4 (57.1)	2 (28.6)		
**Economic status (n (%))**										
Low	7 (36.8)	6 (31.6)	6 (31.6)	0.81	0.70	7 (36.8)	8 (42.1)	4 (21.1)	0.23	0.72
Moderate	16 (29.1)	21 (38.2)	18 (32.7)			15 (27.3)	17 (30.9)	23 (41.8)		
High	7 (41.2)	4 (23.5)	6 (35.3)			8 (47.1)	6 (35.3)	3 (17.6)		
**Job status (n (%))**										
Housewife	7 (25.9)	11 (40.7)	9 (33.3)	0.83	0.76	8 (29.6)	12 (44.4)	7 (25.9)	0.12	0.99
Employment	17 (35.4)	16 (33.3)	15 (31.3)			15 (31.3)	12 (25.0)	21(43.8)		
University student	6 (37.5)	4 (25.0)	6 (37.5)			7 (43.8)	7 (43.8)	2 (12.5)		
**Education status (n (%))**										
Diploma and under	6 (50.0)	4 (33.3)	2 (16.7)	0.36	0.34	2 (16.7)	5 (41.7)	5 (41.7)	0.42	0.49
Bachelor and master	20 (29.9)	25 (37.3)	22 (32.8)			24 (35.8)	20 (29.9)	23 (34.3)		
PhD and above	4 (33.3)	2 (16.7)	6 (50.0)			4 (33.3)	6 (50.0)	2 (16.7)		
**Sleep quality (n (%))**										
Poor	16 (40.0)	12 (30.0)	12 (30.0)	0.44	0.68	15(37.5)	13(32.5)	12(30.0)	0.70	0.94
Good	14 (27.5)	19 (37.3)	18 (35.3)			15 (29.4)	18 (35.3)	18 (35.3)		

All variables are mean ± standard deviation (SD); significant values are shown in bold

*BMI* body mass index, *WC* waist circumference, *WHR* waist- hip ratio, *BFM* body fat mass, *BF* body fat, *SMM* skeletal muscle mass, *FFM* fat free mass, *FMI* fat mass index, *FFMI* fat-free mass index, *SBP* systolic blood pressure, *DBP* diastolic blood pressure, *T* tertile

*P-values* < 0.05 were considered as significant and 0.05, 0.06, and 0.07 were considered marginally significant

*P*.value^*^: obtained from analysis of variance (ANOVA) for continuous variables and chi-square or Fisher exact test for categorical variables

*P*.value^b^: obtained from analysis of covariance (ANCOVA) to adjust potential cofounder (energy intake, age, BMI, physical activity)

There was a marginally significant difference between tertiles of dietary plant protein and the FMI of participants (*P* = 0.05). In addition, individuals with a higher dietary plant protein score had a lower FFMI (*P* = 0.03), and individuals with lower dietary plant scores had greater diastolic blood pressure (DBP) (*P* = 0.04). Moreover, significant and marginally significant differences were observed between tertiles of dietary plant protein and BFM (*P* = 0.03) and %BF (*P* = 0.07) of participants, respectively, after adjustment for confounding factors. Additionally, there was a significant difference between tertiles of dietary plant protein and marital status (*P* = 0.01).

### Dietary food intake across the tertiles of plant and animal protein intake

The dietary intake of all participants according to plant and animal protein tertiles is presented in Table 2. The mean differences in energy intake (*P* =  < 0.05), protein intake (*P* =  < 0.05), carbohydrate intake (*P* =  < 0.05), fat intake (*P* = 0.00), fiber intake (*P* =  < 0.05), PUFA intake (*P* =  < 0.05), monosaturated fatty acid intake (MUFA) intake (*P* = 0.04), vitamin E intake (*P* =  < 0.05), vitamin C intake (*P* =  < 0.05), thiamin intake (*P* = 0.00), vitamin B_6_ intake (*P* =  < 0.05), vitamin B_9_ intake (*P* =  < 0.05), iron intake (*P* = 0.00), zinc intake (*P* =  < 0.05), potassium intake (*P* =  < 0.05) and magnesium intake (*P* =  < 0.05) were statistically significant in the crude model of the tertiles of plant protein. After controlling for energy intake, protein (*P* = 0.04), carbohydrate (*P* =  < 0.05), fat (*P* =  < 0.05), SFA (*P* =  < 0.05), cholesterol (*P* =  < 0.05), thiamin (*P* =  < 0.05), vitamin B_9_ (*P* =  < 0.05), vitamin B_12_ (*P* =  < 0.05), iron (*P* =  < 0.05), potassium (*P* = 0.02) and magnesium (*P* = 0.05) remained significantly different.

**Table 2:** Dietary intake of study participants across the tertiles of plant and animal protein intake

	**Tertiles of plant protein**					**Tertiles of animal protein**					
**Variables**	**T1** ** ≤ 24.81** **(*N* = 30)**	**T2** **24.82- 36.07** **(*N* = 31)**	**T3** ** ≥ 36.08** **(*N* = 30)**	*P-*value^*^	***P-*value**^**b**^	**T1** ** ≤ 21.69** **(*N* = 30)**	**T2** **21.70- 36.32** **(*N* = 31)**	**T3** ** ≥ 36.33** **(*N* = 30)**	*P-*value^*^	***P-*value**^**b**^	
**Nutrient item**											
Energy (kcal/day)	1736.53 ± 387.63	2236.54 ± 400.65	2888.36 ± 774.81	** < 0.05**	-	1953.23 ± 461.48	2346.38 ± 719.79	2558.16 ± 814.20	** < 0.05**	-	
Protein (g/day)	69.53 ± 22.37	85.87 ± 29.43	96.02 ± 22.45	** < 0.05**	**0.04**	64.94 ± 15.86	81.76 ± 18.51	104.86 ± 28.86	** < 0.05**	** < 0.05**	
Carbohydrate (g/day)	235.20 ± 50.93	329.87 ± 77.37	440.90 ± 119.17	** < 0.05**	** < 0.05**	316.31 ± 90.04	340.99 ± 121.15	348.29 ± 145.09	0.56	** < 0.05**	
Fat (g/day)	60.30 ± 19.83	70.98 ± 22.75	87.07 ± 39.83	** < 0.05**	** < 0.05**	50.39 ± 14.17	80.11 ± 34.02	87.55 ± 26.29	** < 0.05**	** < 0.05**	
Dietary fiber (g/day)	16.38 ± 4.07	23.63 ± 8.28	27.49 ± 8.06	** < 0.05**	0.08	18.60 ± 6.19	23.94 ± 8.40	24.94 ± 9.10	** < 0.05**	0.34	
n -3 fatty acids (g/day)	3.12 ± 6.02	1.98 ± 4.63	1.15 ± 0.95	0.22	0.29	1.43 ± 3.44	3.74 ± 6.56	1.02 ± 0.63	**0.03**	0.34	
SFA (g/day)	23.55 ± 10.43	24.49 ± 10.09	28.26 ± 13.87	0.25	** < 0.05**	17.57 ± 5.98	27.77 ± 12.66	30.86 ± 10.84	** < 0.05**	** < 0.05**	
PUFA (mg/day)	13.90 ± 5.63	18.05 ± 7.88	24.59 ± 13.88	** < 0.05**	0.78	13.47 ± 5.29	21.10 ± 11.98	21.87 ± 11.32	** < 0.05**	0.16	
MUFA (mg/day)	664.40 ± 1508.00	219.88 ± 794.22	27.61 ± 13.31	**0.04**	0.14	178.11 ± 620.18	689.86 ± 1557.31	28.26 ± 7.84	**0.02**	**0.02**	
Cholesterol (mg/day)	269.46 ± 113.87	279.90 ± 185.46	258.03 ± 91.38	0.82	** < 0.05**	171.61 ± 61.36	276.93 ± 84.07	358.95 ± 166.81	** < 0.05**	** < 0.05**	
Vitamin C (mg/day)	122.38 ± 47.61	184.49 ± 98.99	178.47 ± 91.40	** < 0.05**	0.10	128.21 ± 58.74	177.89 ± 92.81	179.47 ± 95.27	**0.03**	0.26	
Vitamin E (mg/day)	5.87 ± 2.89	9.98 ± 7.38	11.94 ± 9.78	** < 0.05**	0.45	6.28 ± 3.78	12.13 ± 10.24	9.31 ± 6.29	**0.01**	**0.02**	
Thiamin (mg/day)	1.46 ± 0.28	2.02 ± 0.38	2.62 ± 0.63	** < 0.05**	** < 0.05**	1.86 ± 0.52	2.09 ± 0.67	2.14 ± 0.72	0.20	** < 0.05**	
Vitamin B6 (mg/day)	1.66 ± 0.37	2.26 ± 0.93	2.50 ± 0.73	** < 0.05**	0.15	1.61 ± 0.47	2.19 ± 0.64	2.61 ± 0.88	** < 0.05**	** < 0.05**	
Vitamin B9 (µg/day)	343.57 ± 76.74	500.19 ± 122.63	647.22 ± 151.85	** < 0.05**	** < 0.05**	438.06 ± 143.63	512.68 ± 163.21	539.83 ± 195.63	**0.05**	0.78	
Vitamin B12 (µg/day)	3.91 ± 2.40	3.98 ± 1.96	3.91 ± 1.65	0.98	** < 0.05**	2.32 ± 0.85	4.09 ± 1.46	5.43 ± 2.12	< 0.05	** < 0.05**	
Iron (mg/day)	13.65 ± 3.02	18.73 ± 4.00	24.89 ± 6.28	** < 0.05**	** < 0.05**	18.28 ± 6.79	18.91 ± 6.23	20.07 ± 6.53	0.56	** < 0.05**	
Zinc (mg/day)	9.19 ± 2.59	11.54 ± 3.56	13.67 ± 3.45	** < 0.05**	0.36	8.82 ± 2.30	11.27 ± 2.46	14.31 ± 3.85	** < 0.05**	** < 0.05**	
Potassium (mg/day)	3009.06 ± 688.98	3967.80 ± 1380.14	4237.13 ± 1176.03	** < 0.05**	** < 0.05**	2912.40 ± 795.42	3881.70 ± 1089.92	4422.76 ± 1272.08	** < 0.05**	** < 0.05**	
Magnesium (mg/day)	271.79 ± 61.13	393.01 ± 143.45	374.56 ± 133.64	** < 0.05**	**0.05**	312.60 ± 92.25	374.16 ± 102.42	436.94 ± 167.54	** < 0.05**	0.15	
**Food groups (g/day)**											
Grains	306.19 ± 123.31	410.17 ± 139.19	666.92 ± 283.89	** < 0.05**	** < 0.05**	435.19 ± 133.62	447.64 ± 187.11	498.48 ± 361.45	0.58	0.47	
Nuts and legumes	37.41 ± 18.62	56.28 ± 35.63	81.79 ± 40.51	** < 0.05**	** < 0.05**	47.77 ± 23.41	64.75 ± 44.13	62.66 ± 39.82	0.15		0.49
Fruits	241.41 ± 100.64	372.14 ± 312.31	401.72 ± 219.67	** < 0.05**	0.45	276.05 ± 148.69	334.12 ± 218.62	406.37 ± 306.60	0.10		0.59
Vegetables	292.69 ± 132.15	485.70 ± 316.80	528.40 ± 409.29	** < 0.05**	**0.01**	340.26 ± 156.92	456.70 ± 366.05	510.79 ± 377.58	0.11		0.16
Meats	89.65 ± 65.22	101.77 ± 82.31	81.28 ± 43.18	0.47	**0.01**	40.03 ± 18.49	76.14 ± 24.29	157.38 ± 70.12	** < 0.05**		** < 0.05**
Dairy	323.03 ± 181.07	326.97 ± 191.82	342.38 ± 196.33	0.08	0.16	206.43 ± 100.66	318.76 ± 167.75	467.47 ± 186.79	** < 0.05**		** < 0.05**

All variables are mean ± standard deviation (SD); significant values are shown in bold

*SFA* saturated fatty acids, *PUFA* poly-saturated fatty acids, *MUFA* mono-saturated fatty acids, *T* tertile

*P-values* < 0.05 were considered significant and 0.05, 0.06, and 0.07 were considered marginally significant

*P*.value^*^: obtained from analysis of variance (ANOVA)

*P*.value^b^: obtained from analysis of covariance (ANCOVA) to adjust energy intake

Participants in the highest tertile of animal protein had greater intake of carbohydrates (*P* = 0.03), fat (*P* =  < 0.05), SFAs (*P* =  < 0.05), cholesterol (*P* =  < 0.05), vitamin B_6_ (*P* =  < 0.05), vitamin B_12_ (*P* =  < 0.05), iron (*P* =  < 0.05), zinc (*P* =  < 0.05), and potassium (*P* =  < 0.05) and a lower intake of MUFAs (*P* = 0.02) and vitamin C (*P* = 0.02) after adjusting for confounders. There were no significant differences in the other variables, as shown in Table 2.

### Associations between psychological disorders and dietary plants and animal protein

Table 3 shows the multivariable-adjusted ORs and 95% CIs for the psychological profiles of the plant and animal protein tertiles. There was a significant association between depression (OR_=_ 0.31; 95% CI: 0.10, 0.93; *P*_=_ 0.03), anxiety (OR = 0.20; 95% CI: 0.56, 0.72; *P* = 0.01), and psychological distress (OR = 0.30; 95% CI: 0.10, 0.92; *P* = 0.03) with dietary plant protein in the crude model, which remained significant in Model II (adjusted for energy intake, age, body fat (%), physical activity, marital status, educational status, economic status, and sleep quality) but became marginally significant for depression (OR = 0.27; 95% CI: 0.06, 1.09; *P* = 0.06) and nonsignificant for anxiety (OR = 0.32; 95% CI: 0.07, 1.42; *P* = 0.13) and psychological distress (OR = 0.31; 95% CI: 0.08, 1.25; *P* = 0.10) in the fully adjusted model (adjusted for Model II + dietary intake of total antioxidants, vitamin B group, and PUFA/SFA). However, a marginally positive significant association was observed between animal protein intake and psychological distress in the crude (OR = 2.59; 95% CI: 0.91, 7.34; *P* = 0.07) and adjusted models. A higher intake of dietary animal protein was not significantly related to the risk of depression or anxiety according to the crude and fully adjusted models.

**Table 3:** Crude and multivariable-adjusted odds ratio (95% CI) for depression, anxiety, and stress across tertiles of plant and animal protein int

	**Tertile of plant protein**				**Tertile of animal protein**			
**Variables**	T1^*^ ≤ 24.81 **(*N* = 30)**	**T2** **24.82- 36.07** **(*N* = 31)**	**T3** ** ≥ 36.08** **(*N* = 30)**	***P***_***trend***_	T1^*^ ≤ 21.69 **(*N* = 30)**	**T2** **21.70- 36.32** **(*N* = 31)**	**T3** ** ≥ 36.33** **(*N* = 30)**	***P***_***trend***_
**Depression**		**OR (CI)**	**OR (CI)**			**OR (CI)**	**OR (CI)**	
Crude	1.00	0.55 (0.20, 1.53)	0.31 (0.10, 0.93)	**0.03**	1.00	0.25 (0.08, 0.77)	0.66 (0.24, 1.85)	0.43
Model I^a^	1.00	0.44 (0.15, 1.30)	0.30 (0.09, 0.99)	**0.05**	1.00	0.20 (0.06, 0.68)	0.67 (0.22, 2.07)	0.49
Model II^b^	1.00	0.39 (0.11, 1.37)	0.26(0.06, 0.97)	**0.04**	1.00	0.12 (0.02, 0.55)	0.45 (0.11, 1.75)	0.25
Model III^c^	1.00	0.35 (0.08, 1.46)	0.27 ( 0.06, 1.09)	**0.06**	1.00	0.16 (0.03, 0.76)	0.34(0.07, 1.55)	0.16
**Anxiety**								
Crude	1.00	1.07 (0.39, 2.95)	0.20 (0.56, 0.72)	**0.01**	1.00	0.58 (0.18, 1.81)	1.75 (0.61, 4.97)	0.29
Model I^a^	1.00	1.09 (0.38, 3.15)	0.24(0.06, 0.90)	**0.03**	1.00	0.63 (0.18, 2.13)	3.38 (0.97, 11.79)	**0.05**
Model II^b^	1.00	1.05 (0.30, 3.58)	0.20 (0.04, 0.87)	**0.03**	1.00	0.52 (0.13, 2.06)	3.09 (0.78, 12.15)	0.10
Model III^c^	1.00	1.50 (0.36, 6.10)	0.32 (0.07, 1.42)	0.13	1.00	0.67 (0.15, 2.99)	3.68 (0.75, 18.04)	0.10
**Psychological distress**								
Crude	1.00	0.82 (0.30, 2.25)	0.30 (0.10, 0.92)	**0.03**	1.00	0.50 (0.16, 1.54)	2.59 (0.91, 7.34)	**0.07**
Model I^a^	1.00	0.83 (0.30, 2.34)	0.32 (0.10, 1.04)	**0.05**	1.00	0.47 (0.14, 1.52)	3.13 ( 0.99, 9.89)	**0.05**
Model II^b^	1.00	0.55 (0.15, 1.96)	0.26 (0.07, 0.99)	**0.04**	1.00	0.47 (0.11, 1.91)	3.44 (0.89, 13.30)	**0.07**
Model III^c^	1.00	0.62 (0.16, 2.38)	0.31 (0.08, 1.25)	0.10	1.00	0.59 (0.13, 2.60)	3.87 (0.89, 16.70)	**0.07**

*P-values* < 0.05 were considered significant and 0.05, 0.06, and 0.07 were considered marginally significant

^*^The lowest tertile was considered as a reference group; significant values are shown in bold

^a^Adjusted for energy intake, age, and body fat (%)

^b^Further adjusted for physical activity, marriage status, educational status, economic status, and sleep quality

^c^Additionally adjusted for dietary intake of total antioxidants, vitamin B group, and PUFA/SFA

### Associations between biochemical indicators and plant and animal protein intake

To investigate the relationships between biochemical indicators of plant and animal protein tertiles, we performed a linear regression with crude and adjusted models (Table 4). Our results revealed a negative correlation between plant protein and both KYN (ß = -700.73; 95% CI: -1166.21, -235.26; *P* < 0.001) and IL-6 (ß = -119.96; 95% CI: -209.54, -30.38; *P* < 0.001) (Table 4). Even after adjustment for confounders, the aforementioned associations remained statistically significant (*P* < 0.001).

**Table 4:** B-coefficients (95% CI) for biochemical indicators across tertiles of plant and animal protein intake

	**Tertiles of plant protein**				**Tertiles of animal protein**			
**Variables**	**T1** ** ≤ 24.81** **(*N* = 30)**	**T2** **24.82- 36.07** **(*N* = 31)**	**T3** ** ≥ 36.08** **(*N* = 30)**	**P**_**trend**_	**T1**^**c**^ ** ≤ 21.69** **(*N* = 30)**	**T2** **21.70- 36.32** **(*N* = 31)**	**T3** ** ≥ 36.33** **(*N* = 30)**	**P**_**trend**_
**Kynurenin (nmol/l)**		**ß (CI)**	**ß (CI)**			**ß (CI)**	**ß (CI)**	
Crude	0	-353.47 (-815.17, 108.23)	-700.73 (-1166.21, -235.26)	**0.00**	0	98.05 (-382.62, 578,34)	-144.80 (-629.40, 339.80)	0.55
Model I^a^	0	-371.18 (-832.15, 89.78)	-719.84 (-1201.47, -238.20)	**0.00**	0	151.93 (-329.84, 633.71)	-15.59 (-521.59, 490.39)	0.95
Model II^b^	0	-422.93 (-888.90,43.03)	-721.99 (-1182.44, -261,53)	**0.00**	0	319.16 (-172.57, 810.89)	-16.97 (-519.42, 485.47)	0.94
**Serotonin (ng/ml)**								
Crude	0	-1.35 (-23.32, 20.60)	-7.87 (-30.55, -14.79)	0.49	0	-19.41 (-40.91, 2.08)	-10.91 (-32.48,10.66)	0.32
Model I^a^	0	0.38 (-.21.47, 22.23)	-4.32 (-27.65, 19.00)	0.71	0	-22.58 (-45.03, -2.61)	-16.73 (-38.83, 5.35)	0.13
Model II^b^	0	-3.28 (-25.63, 19.06)	-4.68 (-27.19, -17.83)	0.68	0	-19.15 (-41.45, 3.14)	-17.55 (-39.95, 4.85)	0.12
**Kynurenin/ serotonin**								
Crude	0	-0.06 (-1.33, 1.21)	-0.78 (-2.07, 0.49)	0.23	0	-0.63 (-1.90, 0.64)	0.17 (-1.10, 1.46)	0.78
Model I^a^	0	-0.13 (-1.32, 1.05)	-0.92 (-2.16, 0.31)	0.14	0	-0.24 (-1.42, 0.94)	0.94(-0.30, 2.18)	0.13
Model II^b^	0	-0.08 (-1.27, 1.11)	-0.94 (-2.12, 0.24)	0.11	0	-0.04 (-1.25, 1.16)	1.10 (-0.13, 2.33)	**0.07**
**IL-6 (ng/l)**								
Crude	0	-7.33 (-15.58, 0.92)	-11.99 (-20.32, -3.67)	**0.00**	0	4.44 (-4.07, 12.95)	-1.82 (-10.40, 6.75)	0.69
Model I^a^	0	-7.69 (-15.96, 0.58)	-12.23 (-20.88, -3.59)	**0.00**	0	5.29 ( -3.26, 13.84)	0.23 (-8.75, 9.22)	0.92
Model II^b^	0	-7.58 (-16.08, 0.91)	-11.63( -20.09, -3.16)	**0.00**	0	7.24 (-1.21, 15.70)	-1.36 (-10.24, 7.50)	0.76

*P*-values < 0.05 were considered significant and 0.05, 0.06, and 0.07 were considered marginally significant

^a^Adjusted for energy intake, age, and body fat (%)

^b^Further adjusted for physical activity, marriage status, educational status, economic status, and sleep quality

^c^Lowest tertile was considered as a reference group; significant values are shown in bold

Additionally, we found a marginally significant positive association between the KYN/serotonin ratio and animal protein intake in Model II (ß = 1.10; 95% CI: -0.13, 2.33; *P* = 0.07). No significant difference was found between other biochemical indicators and animal protein consumption.

### The association between the abundance of bacteria and plant and animal protein intake

As shown in Table 5, the findings revealed a marginally significant negative relationship between the abundance of *Firmicutes* (β = -1.17 × 10^11^, SE = 6.11 × 10^10^, *P* = 0.05) and plant protein intake, which became significant in all adjusted models (*P* < 0.05). Additionally, our results showed a significant negative association between the *Firmicutes/Bacteroidetes* ratio and dietary plant protein after controlling for confounding factors (Model III: β = -1.27 × 10^3^, SE = 5.99 × 10^2^, *P* = 0.03).

**Table 5:** B-coefficients (SE) for the abundance of bacteria across tertiles of plant and animal protein

	**Tertiles of plant protein**				**Tertiles of animal protein**			
**Variables**	**T1** ** ≤ 24.81** **(*N* = 30)**	**T2** **24.82- 36.07** **(*N* = 31**)	**T3** ** ≥ 36.08** **(*N* = 30)**	***P*-value**	**T1**^**d**^ ** ≤ 21.69** **(*N* = 30)**	**T2** **21.70- 36.32** **(*N* = 31)**	**T3** ** ≥ 36.33** **(*N* = 30)**	***P*-value**
**Bacteria**								
*Akkermansia –muciniphila* (CFU/gr)		**ß (SE)**	**ß (SE)**			**ß (SE)**	**ß (SE)**	
Crude	0	-3.56 × 10^11^ (5.27 × 10^11^)	-7.69 × 10^11^ (5.03 × 10^11^)	0.12	0	-9.95 × 10^10^ (3.61 × 10^11^)	-1.32 × 10^10^ (3.64 × 10^11^)	0.97
Model I^a^	0	-3.96 × 10^11^ (5.27 × 10^11^)	-5.73 × 10^11^ (5.16 × 10^11^)	0.26	0	-1.06 × 10^11^ (3.55 × 10^11^)	6.55 × 10^10^ (3.72 × 10^11^)	0.86
Model II^b^	0	-7.07 × 10^11^ (5.05 × 10^11^)	-6.00 × 10^11^ (4.64 × 10^11^)	0.19	0	-4.23 × 10^10^ (3.46 × 10^11^)	-2.70 × 10^9^ (3.64 × 10^11^)	0.99
Model III^c^	0	-6.08 × 10^11^ (5.16 × 10^11^)	-5.46 × 10^11^ (4.66 × 10^11^)	0.24	0	-3.33 × 10^10^ (3.46 × 10^11^)	1.83 × 10^10^ (3.64 × 10^11^)	0.96
*Fecalibacterium -parausnitzii* (CFU/gr)								
Crude	0	9.74 × 10^17^(4.30 × 10^18^)	5.74 × 10^18^(4.33 × 10^18^)	0.68	0	3.64 × 10^18^ (4.32 × 10^18^)	4.10 × 10^17^ (4.36 × 10^18^)	0.92
Model I^a^	0	-7.11 × 10^17^(4.00 × 10^18^)	3.22 × 10^18^(4.20 × 10^18^)	0.44	0	2.61 × 10^18^ (4.01 × 10^18^)	-3.65 × 10^18^ (4.21 × 10^18^)	0.38
Model II^b^	0	-2.68 × 10^18^(4.30 × 10^18^)	2.98 × 10^18^(4.07 × 10^18^)	0.46	0	4.97 × 10^18^ (4.01 × 10^18^)	-3.57 × 10^18^ (4.21 × 10^18^)	0.39
Model III^c^	0	-3.26 × 10^18^(4.09 × 10^18^)	1.55 × 10^18^(4.14 × 10^18^)	0.70	0	5.24 × 10^18^ (3.97 × 10^18^)	-3.10 × 10^18^ (4.17 × 10^18^)	0.45
*Prevotella* (CFU/gr**)**								
Crude	0	-7.61 × 10^18^(4.12 × 10^18^)	2.98 × 10^18^(4.07 × 10^18^)	0.25	0	-1.26 × 10^19^ (4.85 × 10^18^)	-1.04 × 10^19^ (4.89 × 10^18^)	0.03
Model I^a^	0	-7.31 × 10^18^(4.73 × 10^18^)	-7.61 × 10^18^(4.97 × 10^18^)	0.12	0	-1.19 × 10^19^ (4.68 × 10^18^)	-9.82 × 10^18^(4.91 × 10^18^)	0.04
Model II^b^	0	-8.82 × 10^18^(4.95 × 10^18^)	-7.03 × 10^18^(4.89 × 10^18^)	0.15	0	-9.72 × 10^18^ (4.84 × 10^18^)	-8.70 × 10^18^ (5.08 × 10^18^)	0.08
Model III^c^	0	-8.36 × 10^18^(4.94 × 10^18^)	-5.89 × 10^18^(5.01 × 10^18^)	0.24	0	-9.97 × 10^18^ (4.79 × 10^18^)	-9.20 × 10^18^ (5.04 × 10^18^)	**0.06**
**Phylum**								
*Firmicutes*								
Crude	0	-1.04 × 10^11^(6.06 × 10^10^)	-1.17 × 10^11^(6.11 × 10^10^)	0.05	0	-6.48 × 10^10^(6.16 × 10^10^)	1.37 × 10^9^(6.21 × 10^10^)	0.98
Model I^a^	0	-9.77 × 10^10^(5.94 × 10^10^)	-1.50 × 10^11^(6.24 × 10^10^)	0.01	0	-5.84 × 10^10^(6.17 × 10^10^)	-4.62 × 10^9^(6.47 × 10^10^)	0.94
Model II^b^	0	-1.07 × 10^11^(6.01 × 10^10^)	-1.56 × 10^11^(5.95 × 10^10^)	0.009	0	-7.22 × 10^10^(6.11 × 10^10^)	-1.30 × 10^10^(6.41 × 10^10^)	0.83
Model III^c^	0	-1.08 × 10^11^(6.03 × 10^10^)	-1.60 × 10^11^(6.12 × 10^10^)	**0.009**	0	-7.30 × 10^10^ (6.11 × 10^10^)	-1.47 × 10^10^(6.42 × 10^10^)	0.81
*Bacteroidetes*								
Crude	0	-6.37 × 10^11^(8.54 × 10^11^)	7.24 × 10^11^(8.61 × 10^11^)	0.40	0	5.94 × 10^11^(8.60 × 10^11^)	-3.64 × 10^11^(8.67 × 10^11^)	0.67
Model I^a^	0	-9.35 × 10^11^(8.00 × 10^11^)	2.03 × 10^11^(8.40 × 10^11^)	0.80	0	4.84 × 10^11^(8.06 × 10^11^)	-8.79 × 10^11^(8.45 × 10^11^)	0.29
Model II^b^	0	-1.43 × 10^12^(8.20 × 10^11^)	2.77 × 10^9^(8.11 × 10^11^)	0.99	0	3.06 × 10^11^(8.07 × 10^11^)	-1.06 × 10^12^(8.46 × 10^11^)	0.20
Model III^c^	0	-1.49 × 10^12^(8.20 × 10^11^)	-1.54 × 10^11^(8.32 × 10^11^)	0.85	0	3.33 × 10^11^(8.04 × 10^11^)	-1.01 × 10^12^(8.45 × 10^11^)	0.23
*Fir/ Bac*^e^								
Crude	0	-7.94 × 10^2^(6.24 × 10^2^)	-7.95 × 10^2^(6.34 × 10^2^)	0.21	0	-4.75 × (6.29 × 10^2^)	7.74 × 10^2^(6.29 × 10^2^)	0.21
Model I^a^	0	-7.62 × 10^2^(6.01 × 10^2^)	-1.26 × 10^3^(6.41 × 10^2^)	0.04	0	3.88 × 10^1^ (6.25 × 10^2^)	5.92 × 10^2^(6.48 × 10^2^)	0.36
Model II^b^	0	-1.03 × 10^3^(5.75 × 10^2^)	-1.34 × 10^3^(5.81 × 10^2^)	0.02	0	2.09 × 10^2^ (5.87 × 10^2^)	5.58 × 10^2^(6.02 × 10^2^)	0.34
Model III^c^	0	-1.04 × 10^3^(5.77 × 10^2^)	-1.27 × 10^3^(5.99 × 10^2^)	**0.03**	0	1.90 × 10^2^ (5.82 × 10^2^)	5.23 × 10^2^(5.98 × 10^2^)	0.38

*P-values* < 0.05 were considered significant and 0.05, 0.06, and 0.07 were considered marginally significant

^a^Adjusted for energy intake, and age

^b^Further adjusted for physical activity, marriage status, educational status, socioeconomic status, and sleep quality

^c^Additionally adjusted for body fat percentage (%BF)

^d^Lowest tertile was considered as a reference group; significant values are shown in bold

^e^Firmicutes/Bacteriodetes

Furthermore, a significant negative correlation between dietary animal protein and *Prevotella* abundance was detected in the crude model (β = -1.04 × 10^19^, SE = 4.89 × 10^18^, *P* = 0.03), which remained marginally significant in the fully adjusted model (β = -9.20 × 10^18^, SE = 5.04 × 10^18^, *P* = 0.06).

## Discussion

To our knowledge, this study is the first to investigate the associations between animal and plant protein intake and mental health parameters (depression, anxiety, and psychological stress), tryptophan metabolite pathways, and the abundance of some gut microbiota in a sample of healthy Iranian women. Our findings showed that increased plant protein intake is inversely associated with depression, anxiety, and stress. Individuals with high animal protein intake, on the other hand, were more likely to experience symptoms of stress but not depression or anxiety. The mentioned relationships were independent of probable confounders related to lifestyle, anthropometric measurements, and dietary intake. We also found an inverse relationship between greater plant protein intake and levels of KYN and IL-6 and a marginally significant direct association between animal protein intake and the KYN/Serotonin ratio. A greater consumption of plant and animal foods affected the abundance of some gut bacteria and phyla. The results of this study revealed no meaningful associations between protein sources and other gut bacteria or other variables, probably due to uncontrolled confounding variables and a lack of evaluation of the dose–response association between dietary protein intake and the studied variables.

In recent years, several studies have focused on the effects of dietary proteins on mental health [21, 22]. However, the results are contradictory, and no study has investigated the effects of plant and animal proteins on the aforementioned outcomes jointly; therefore, our findings provide novel insight into this matter. Due to the widespread prevalence of mental disorders, their effects on quality of life, and the economic burden on societies [23–26],mental disorders have received increasing attention, and several predictive factors that are potential markers of mental illness have been identified [27–29] to identify the most practical approach for better mood state and psychological disorder management. Diet seems to play a critical role, and protein, provided by both animal and plant food sources, is one of the important components related to mental well-being [29–31]. In one study, adult women who consumed high amounts of animal protein demonstrated anxiety, stress, and depressive behavior [21]. Furthermore, a clinical study revealed that restricting the consumption of animal-based foods has beneficial effects on short-term mood [31]. Jin Y et al. conducted a cross-sectional study among 892 Asian residents of the United States and suggested an inverse association between a vegetarian diet and the prevalence of depression [32]. The same conclusions were shown in two different cohort studies between depression and the Mediterranean diet, characterized by high plant-derived foods and low red meat intake [33], as well as the dietary approach to hypertension (DASH) diet [34]. Another inverse relationship was found between healthy dietary patterns, including fruits, vegetables, nuts, and legumes, and anxiety and depressive symptoms [35, 36]. In contrast to our results, a meta-analysis revealed that meat consumption may be related to a greater risk of depression [22]. Another study revealed no significant association between increased plant protein intake and mental disorders [21]. Interestingly, one publication indicated that the intake of protein from milk and other milk products was inversely related to depressive symptoms [37]. a-Lactalbumin, which is a food- based rich source of tryptophan [38], has been proposed to have beneficial effects on mood [39]. Given that mental disorders are the result of neurochemical imbalances, it has been suggested that serotonin imbalance can lead to depression [40]. In other words, tryptophan, the primary precursor of serotonin [41], is constantly in competition with other amino acids (leucine, isoleucine, phenylalanine, valine, and tyrosine) to bind the carrier and transport through the blood‒brain barrier [42] to have positive effects on brain and mental status. Interestingly, individuals who consumed plant-derived meals compared to animal protein consumers were reported to have higher folate, brain tryptophan, and tyrosine levels, and a rich source of folate was reported by plant-based food intake [43, 44]; therefore, it appears that greater tryptophan, folate, and tyrosine levels provide enough serotonin and other neurotransmitters to affect mental status and improve mood [29, 45–47]. Excessive homocysteine levels are another putative mechanism for neurotransmitter deficiency [48] and major psychological disorders [49]. Homocysteine is converted from methionine, which is an abundant amino acid in red meat. Its elevated level is the result of high animal food consumption and is more common in women with insufficient folate concentrations [50].

In agreement with our results, one study revealed that the consumption of plant-based foods such as fruits and vegetables could mitigate the levels of IL-6 and other inflammatory markers [51]. Compared to an omnivorous diet, a vegetarian diet is also linked to lower serum C-reactive protein (CRP) [52]. An observational study among women concluded that vegetarians had lower concentrations of KYN than omnivores, and no significant relationships were observed with inflammation biomarkers [53]. In one cohort study, the Mediterranean diet was linked to lower levels of KYN [54]. The exact mechanisms underlying the protective effects of plant-derived foods are still unknown. Plant-based diets contain essential nutrients, dietary fiber, antioxidants, polyphenols [55–58], and other components, such as vitamin C, folate, and magnesium, which appear to reduce oxidative stress and inflammation [59–63]. Additionally, lower levels of KYN might contribute to better liver status and lower plasma TRP. A lower alanine transaminase (ALT) level among vegetarians is an indicator of better liver function, which may accelerate dietary TRP metabolism in the hepatic KYN pathway and decrease the KYN concentration [64]. In the human gut, Bacteroidetes and Firmicutes constitute more than 90% of the microbiota [65]; therefore we addressed these two major bacterial phyla with species in the present study. The gut bacterial composition is considered relatively stable; nevertheless, it could be influenced by diet. The effects of dietary proteins, especially plant proteins, on the gut microbiota were first investigated in 1977 [66]; thus, few studies have been conducted on these effects. In the present study, our data showed a lower abundance of *Firmicutes/Bacteroidetes* and a significantly lower abundance of *Firmicutes* with greater plant protein intake. The ratio of *Firmicutes* to *Bacteroidetes* may be a putative biomarker for inflammation, and plant proteins modulate the immune response through this reduction [67, 68]. On the other hand, a borderline significant association between increased consumption of animal protein and decreased abundance of *Prevotella* was observed. Indeed, *Prevotella*, belonging to the phylum *Bacteroidetes*, is hypothesized to be linked to higher intake of plant proteins, carbohydrates, and fiber: rich food compositions of the Mediterranean diet [69]. Notably, greater plant protein intake, by improving and regulating the composition of gut microbiota could in turn have health-promoting effects and lower the risk of obesity and its related disorders [70–72]. Although several studies have reported a positive correlation between protein intake and total microbial diversity [73–75], Zhu et al. reported that, in comparison with rats fed soy protein, rats fed meat proteins for a short time had a greater diversity of gut bacteria. They also explained that the two most predominant phyla in rats in response to different dietary proteins were *Firmicutes* and *Bacteroidetes*. Furthermore, rats fed soy protein had a greater abundance of *Bacteroidetes* [76]. Another study among overweight people evaluating the impact of the amount and source of dietary protein (plant and animal proteins) supplementation on metabolite production in the gut microbiota indicated no significant difference in bacterial diversity or composition between intervention groups [77]. In high-fiber, plant-derived foods, an elevated ratio of *Bacteroidetes/Firmicutes* [78] and improved intestinal flora composition [79] were observed. The growth of beneficial gut microbiota populations may lead to bioactive metabolite production [66]. Another study, however, detected no significant change in the *Bacteroidetes/Firmicutes* ratio after following a low-fat vegan diet for 16 weeks, despite an increase in the abundance of *Bacteroidetes* [80]. As such, shifting toward plant-based protein intake may be associated with better mental outcomes through decreasing inflammatory markers, influencing the TRP metabolite pathway, lowering kynurenine concentration, regulating the gut microbiota, and improving dysbiosis.

The current study could broaden our knowledge in terms of plant and animal protein intake, mental health, tryptophan metabolite pathways, and the gut microbiota. Several potential confounders were adjusted, and a valid FFQ was applied to assess most of the food items and classify food groups. Nevertheless, there are several limitations to be considered. First, the cross-sectional design of this study prevented us from reaching a causal-effect relationship. Another limitation is that this study was conducted on apparently healthy women aged 18–50 years. Consequently, the findings might not be generalizable to other populations with different genders, ages, and health conditions. Moreover, different seasons, which are reported to influence depressive symptoms, were not considered in this study [81]. Finally, the DASS-21 is a self-reported screening instrument to measure the severity of depression, anxiety, and stress, not for depression and anxiety diagnoses.

## Conclusion

In conclusion, we found inverse associations between high plant protein and psychological disorders, KYN levels, and inflammatory markers, while higher animal protein was associated with increased psychological stress and KYN/serotonin concentrations. In addition, a greater consumption of plant protein is related to a lower abundance of *Firmicutes* and a lower *Firmicutes/Bacteroidetes* ratio, and a diet high in animal protein is correlated with a lower abundance of *Prevotella*. However, further studies are needed to better elucidate the associations between dietary protein intake and these outcomes.

## Materials and methods

### Participant

Ninety-one healthy women living in Tehran, Iran, were randomly assigned to the present cross-sectional study through community-based sampling, which included recruitment through social media, municipal health centers, and phone calls. To participate in the study, women who met the following inclusion criteria were included: had a body mass index (BMI) between 20 and 35 kg/m2, were aged 18–50 years, were in their luteal phase, and expressed a willingness to participate. Subjects suffering from any disease or health problem based on patient reports and medical history (cardiovascular disease, liver, kidney, thyroid, cancer, diabetes, heart failure, neurological or mental diseases, and acute or chronic infections) were excluded. Furthermore, subjects who consumed any medication affecting the gut microbiota composition, such as antibiotics and laxatives, during the last 3 months; who were taking weight loss medication and supplements; who were taking neuropsychiatric drugs such as antidepressants and sedatives; who were consuming supplementary vitamins and minerals except vitamin D; who were using commercial probiotics or fiber products within the previous 3 months; or who were using cigarettes, tobacco, drugs, or alcohol were excluded. Additionally, women who were pregnant, lactating, or in the postmenopausal phase and who had been using oral contraceptives (OCP) within the last 3 months were excluded from the study. The same applied to professional athletes, individuals who had undergone weight loss surgeries or recent weight changes, and those following special diets such as ketogenic or vegetarian diets. All study participants were clearly informed of their right to withdraw at any point and were required to provide written informed consent before the start of the research. The study protocol, including the consent process, was thoroughly reviewed and approved by the Ethics Committee of Tehran University of Medical Sciences, as evidenced by the ethics code (IR.TUMS.MEDICINE.REC.1401.168).

### General information of participants

General data on age, education, occupation, menopausal status, marital status, and medication history were collected through face-to-face interviews. The Pittsburgh Sleep Quality Index (PSQI) was used to estimate the subjective sleep quality of the participants [82]. The PSQI produces scores ranging from 0 to 21, with a score of 5 or higher indicating poor sleep quality [82].

### Anthropometric and body composition assessment

Participants were weighed without shoes and in minimal clothing using a calibrated digital scale (SECA, Humburg, Germany). Heights were measured while standing normally with a nonelastic tape to within 0.5 cm. The weight was divided by the square of the subject’s height (in square meters) (kg/m2) to calculate BMI. A bioelectrical impedance analyzer (BIA) from Inbody 770 Co., Seoul, Korea, was used to evaluate body composition. It was conducted safely and according to the manufacturer’s instructions [83]. To obtain different measurements, such as body composition, waist circumference (WC), hip circumference (HC), waist-to-hip ratio (WHR), body fat mass (BFM), fat-free mass index (FFMI), and fat mass index (FMI), the BIA used an electric signal that passed through the palms and soles of the bare feet.

### Physical activity and blood pressure assessment

Using the International Physical Activity Questionnaire (IPAQ), a tool created by the World Health Organization (WHO), levels of physical exercise were estimated [84]. The mentioned tool was previously evaluated for validity and reliability and was found to be suitable for Iranian adult women [85]. After using standard tables to calculate minutes spent on different physical activities, metabolic equivalent (MET) amounts were recorded. Data were entered into the relevant Excel file, and subjects’ physical activity levels were calculated using MET/min/week. Scores will be estimated based on the frequency and duration of mild, moderate, high, and extremely high-intensity activities using a list of common daily activities.

Using an OMRON (manufactured in Germany) blood pressure monitor, the systolic and diastolic blood pressure was measured from the left arm while the subjects were seated and rested for five minutes.

### Dietary intake assessment

A dietary intake assessment was conducted over the previous 12 months utilizing a 147-item semiquantitative FFQ [86]. The validity and reliability of the FFQ have been proven previously [87]. In a face-to-face interview, a trained dietician asked respondents to record their daily, weekly, or monthly food intake over the last year. Using common household measurements, the portion sizes were converted to grams. The category of animal protein refers to the sum of meat (beef, lamb), poultry, fish, egg, and dairy intake of participants. Whole and refined grains, legumes, nuts and seeds, fruits, and vegetables were the components that made up the plant protein group. Dietary consumption data were imported and evaluated with Nutritionist IV food analyzer software to estimate macronutrient, micronutrient, and total energy intake. The software was designed by the Hearst Corporation in San Bruno, California [88].

### Biochemical and laboratory assessment

Following the overnight fasting period, all participants provided a morning venous blood sample between 8:00 and 10:00 am for biochemical analysis. This blood sample was then centrifuged, split into smaller aliquots, and kept at -80 °C for further examination. The serum levels of KYN, serotonin, and IL-6 were determined using the enzyme-linked immune sorbent assay (ELISA) technique (Crystal Day Biotech Co., Ltd.) following the manufacturer’s instructions for each kit. All samples were analyzed using established protocols at the Nutrition and Biochemistry Laboratory, School of Nutritional Sciences and Dietetics, TUMS.

### DNA extraction and gut microbiota analysis

DNA extraction and 16S rRNA gene sequencing were conducted following established guidelines. DNA extraction was carried out using the FavorPrep™ Stool DNA Isolation Mini Kit from Favorgen Biotech based on the manufacturer’s instructions. The purity and concentration of the extracted DNA were determined using agarose gel electrophoresis and a NanoDrop ND8000 (Thermo Scientific, USA). The purified DNA was stored at -20 °C before amplification. PCR primers were constructed to determine the abundances of bacterial phyla (*Firmicutes* and *Bacteroidetes*), genera, and species (*Fecalibacterium prausnitzii*, *Akkermansia muciniphila*, *Prevotella,* and *E. coli*) (Table 6). The qPCR mixture contained 5 μl of SYBR Premix Ex Taq II (RR820L; Takara, Japan), 0.5 μl of each of the specific 16S rRNA primers, 1 μl of template DNA and 3 μl of water. This qPCR was conducted under the following conditions: initial denaturation for 4 min at 94 °C, 40 cycles of denaturation at 95 °C for 30 s, annealing at 58 °C for 45 s, and a final extension at 72 °C for 45 s. Every reaction was carried out in duplicate. To quantify the bacterial mass for each fecal sample, the cycle threshold (CT) was acquired from serial dilutions of DNA from the standard strain *Escherichia. Coli* (ATCC 25922) with known DNA concentrations on a standard curve. By utilizing the logarithm of CT and the DNA concentration, a semilog regression line graph was constructed for a standard curve.

**Table 6:** Primers sequencing and annealing temperature used in the present study

Target bacteria	sequencing	Tm	Reference
*E.coli*	Forward: CATTGACGTTACCCGCAGAAGAAGC	55 °C	[89]
	Reverse: CTCTACGAGACTCAAGCTTGC		
*Prevotella*	Forward: CACCAAGGCGACGATCA	55 °C	[90]
	Reverse: GGATAACGCCYGGACCT		
*Faecalibacterium prausnitzii*	Forward: GGAGGAAGAAGGTCTTCGG	55 °C	[91]
	Reverse: AATTCCGCCTACCTCTGCACT		
*Akkermansia muciniphila*	Forward: CAGCACGTGAAGGTGGGGAC	55 °C	[89]
	Reverse: CCTTGCGGTTGGCTTCAGAT		
*Firmicutes*	Forward: GGAGYATGTGGTTTAATTCGAAGCA	60 °C	[92]
	Reverse: AGCTGACGACAACCATGCAC		
*Bacteroidetes*	Forward: AAACTCAAAKGAATTGACGG	55 °C	[91]
	Reverse: GGTAAGGTTCCTCGCGCTAT		

*Tm* temperature

### Assessment of the psychological profile

To evaluate the psychological profile, a questionnaire containing the Depression, Anxiety, and Stress Scale (DASS-21) was administered. The reliability of this questionnaire has been established previously. The three DASS subscales each include 7 questions with four answer options, scored from 0 (never applied to me) to 3 (applied to me very much or most of the time). The total score was calculated by summing the scores of each of the three subscales and then multiplying by two. The results were categorized as normal, mild, moderate, severe, or extremely severe for each subscale. For the statistical analysis (logistic regression), the subjects were categorized into normal and abnormal groups.

### Statistical analysis

Participants were classified into tertiles of dietary plant and animal protein intakes.. To compare participant characteristics among tertiles of plant and animal protein, one-way analysis of variance (ANOVA) was used for continuous variables, and the chi-square test or Fisher’s exact test was used for categorical data. For quantitative variables, data are represented as the mean and standard deviation (SD), whereas categorical variables are represented as numbers (percentages). By controlling for age, BMI, physical activity, and total calorie consumption, analysis of covariance (ANCOVA) was utilized to display all characteristics among tertiles of plant and animal protein intake. Participants’ dietary intakes were compared through ANOVA, and ANCOVA was used to compare caloric intake among plant and animal protein tertiles. Binary logistic regressions were applied to determine odds ratios (ORs) and 95% confidence intervals (CIs) for psychological profiles according to plant and animal protein tertiles in both crude and multivariable-adjusted models. Adjustments were made for energy intake, age, body fat percentage (%BF), physical activity, marital status, educational level, economic situation, and sleep quality. To find an independent relationship with dietary intake, in the last model, dietary intake possibly related to psychological disorders [6, 7, 28], consisting of fatty acid type [(poly-saturated fatty acid (PUFA)/saturated fatty acid (SFA)], vitamin B group (B_1_, B_2_, B_6_, folate, pantothenic acid), and total antioxidants (vitamins C, E, selenium, beta carotene), was also evaluated.

**Fig. 2:**
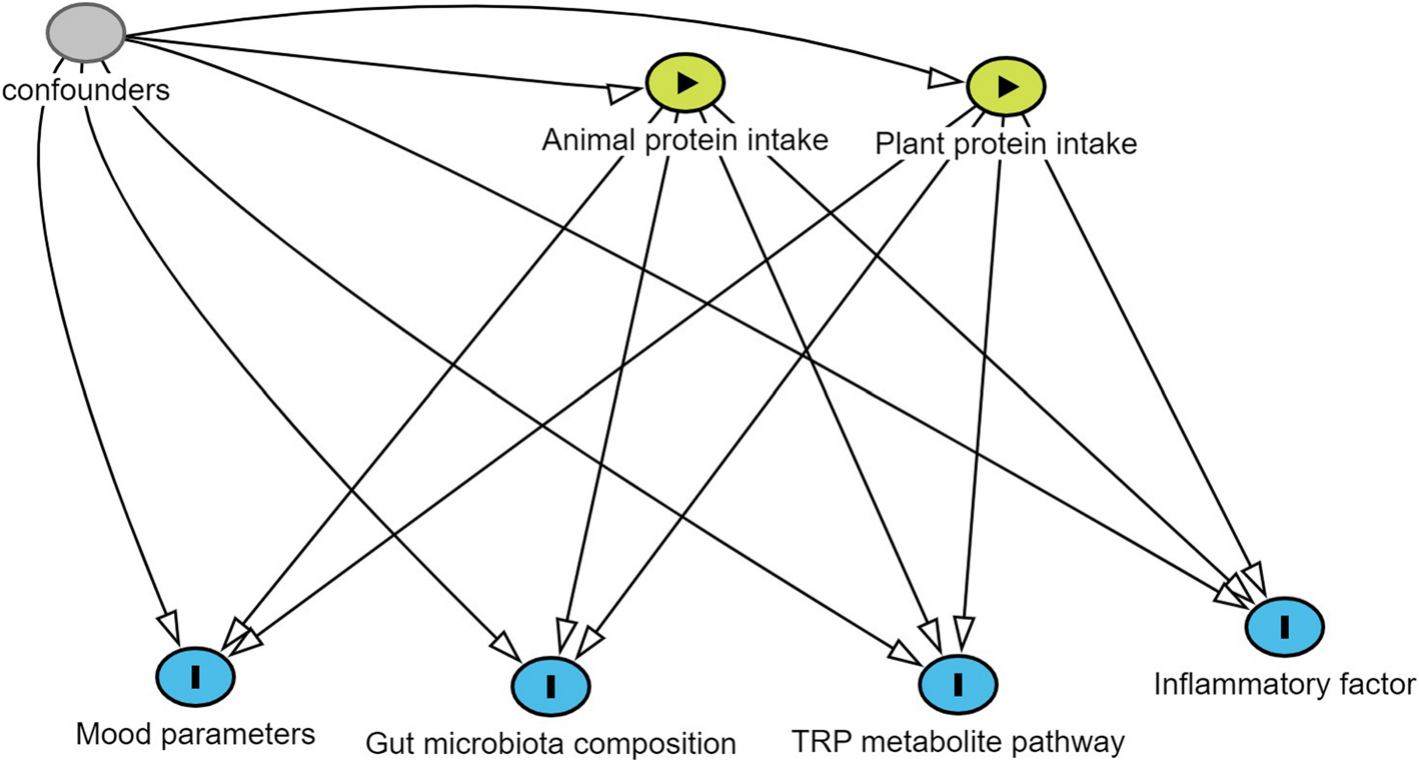
Directed acyclic graph (DAG) for the association between study variable. Green circles: exposures, Blue circles: outcomes, Gray circles: confounders, Arrows: associations. Mood parameters: depression, anxiety, stress; Gut microbiota composition: Akkermansia, Prevotella, Fecalibacterium, Bacteriodetes, Firmicutes; TRP metabolite pathway: kynurenine, serotonin; Inflammatory factor: IL-6

Linear regression models applying GLM were used to analyze the relationships of plant and animal protein with KYN, serotonin, and IL-6 and the abundances of certain bacteria and the Firmicutes and Bacteroidetes phyla. All associations were provided in both crude and adjusted models for confounding variables, such as energy intake, age, physical activity, marital status, educational level, economic status, sleep quality, and %BF. To obtain the trend of B-coefficients and odds ratios (ORs) across categories of dietary protein intakes, tertiles of intake were considered as an ordinal variable. In all models, subjects in the first dietary plant and animal protein intake category were considered the reference category. All reported *P*-values less than 0.05 were considered significant, whereas 0.05, 0.06, and 0.07 were marginally significant. Figure 2 illustrates the links between exposure, outcomes, and confounding factors in a structured network format.

## Data Availability

The research data sets used and/or analyzed in the current study are accessible from Khadijeh Mirzaei upon reasonable request.

## Abbreviations

TRP: Tryptophan
FFQ: Food frequency questionnaire
DASS-21: Depression Anxiety Stress Scales
qPCR: Real-time quantitative polymerase chain reaction
KYN: Kynurenine
GLM: Generalized linear model
IDO: Indoleamine-2, 3-dioxygenase
BMI: Body mass index
OCP: Oral contraceptives
PSQI: Pittsburgh Sleep Quality Index
BIA: Bioelectrical impedance analyzer
WC: Waist circumference
HC: Hip circumference
WHR: Waist-to-hip ratio
BFM: Body fat mass
FFMI: Fat-free mass index
FMI: Fat mass index
IPAQ: International Physical Activity Questionnaire
WHO: World Health Organization
MET: Metabolic equivalent
ELISA: Enzyme-linked immune sorbent assay
ANOVA: One-way analysis of variance
SD: Standard deviation
ANCOVA: Analysis of covariance
ORs: Odds ratios
CIs: Confidence intervals
%BF: Body fat percentage
PUFA: Poly-saturated fatty acids
SFA: Saturated fatty acids
DBP: Diastolic blood pressure
MUFA: Mono-saturated fatty acids
CRP: C-reaction protein
ALT: Alanine- amino transfers
